# Postoperative Atrial Fibrillation Among Patients Undergoing Isolated Coronary Artery Bypass Grafting

**DOI:** 10.7759/cureus.4333

**Published:** 2019-03-27

**Authors:** Ali R Mangi, Kashif Zia, Taimur A Ali, Musa Karim, Saulat H Fatimi

**Affiliations:** 1 Cardiac Surgery, National Institute of Cardiovascular Diseases (NICVD), Karachi, PAK; 2 Miscellaneous, National Institute of Cardiovascular Diseases (NICVD), Karachi, PAK; 3 Cardiothoracic Surgery, The Aga Khan University, Karachi, PAK

**Keywords:** coronary artery bypass grafting, postoperative atrial fibrillation

## Abstract

Introduction

Postoperative atrial fibrillation (AF) is the commonest of all the known cardiac arrhythmias after cardiac surgery. The postoperative AF has both short- and long-term adverse impacts on patients, like prolonged intensive care unit (ICU) stay, increased frequency of reoperations, myocardial infarction, increased use of inotropes, and intra-aortic balloon pump (IABP). There is a paucity of data regarding the postoperative AF after isolated coronary artery bypass grafting (CABG) and its risk factors in our geographic location. Therefore, the aim of this study was to determine the frequency of postoperative atrial fibrillation among patients undergoing isolated CABG at a tertiary care hospital of Karachi, Pakistan.

Methods

This prospective observational study was conducted on 163 consecutively selected patients undergoing first time isolated CABG at the Department of Cardiothoracic Surgery, Aga Khan University Hospital, Karachi. Patients with redo-sternotomy, preoperative atrial fibrillation and with other cardiac pathology were excluded from the study. Postoperative AF was defined in the patients with postoperative 12-lead electrocardiographic (ECG) finding of absence of P waves, replaced by unorganized electrical activity and irregular R-R intervals. Data analysis was carried out using IBM SPSS Statistics for Windows, Version 21.0 (IBM Corp., Armonk, NY, USA).

Results

A total of 163 patients were enrolled with the mean age of 58.66 ± 9.77 years ranging between 40 and 85 years with male predominance of 81% (132). The most common comorbidity was hypertension in about 68.1% (111), followed by diabetes mellitus in 54.6% (89) patients. Postoperative AF was observed in 42 (25.8%) patients. Most of the patients who developed postoperative AF, were overweight with mean body mass index (BMI) of 27.04 ± 4.85 kg/m^2^, 76.2% (32) had a history of hypertension, diabetes mellitus was associated with 33.3% (14) patients with postoperative AF and 50.0% (21) of them were smokers. Distribution of coronary artery disease in patients with postoperative AF was observed as three vessels coronary artery disease (3VCAD) in 83.3% (35), two-vessel coronary artery disease (2VCAD) was present in 7.1% (three), and rest of 9.5% (four) patients had single-vessel coronary artery disease (SVCAD).

Conclusion

The frequency of postoperative atrial fibrillation in our study was found to be 25.9% which is comparable to world literature. An important finding that comes through this study is a younger population undergoing CABG, which raises the possibility of early manifestation of ischemic heart disease in our region. This, however, needs further investigation. We were unable to point out the factors predictive of postoperative AF; studies with larger sample size would help in that regard.

## Introduction

In developed countries, coronary artery disease (CAD) is the leading cause of mortality. CAD may result from the combination of ancient genes with sedentary lifestyles, in which there is a high tendency to eat the high-calorie diet in large quantity. In the modern world, this is further supported by fatty snacks and motorized transport [1]. Coronary artery disease results from atheromatous changes in coronary arteries secondary to the range of diseases. In recent years the explanatory paradigm of CAD has changed because it was identified that coronary plaques exist from stable plaque containing poor lipid with a thick fibrous cap to the unstable plaque with lipid-rich material and thin fibrous cap [2]. Unstable plaques are more susceptible to rupture. As it ruptures, the consequent release of prothrombotic and vasoconstrictive factors increases and results in complete occlusion of the coronary artery. Clinical outcome is dependent on the balance between the prothrombotic and thrombolytic pathways of the body at the rupture site [3]. Transient occlusion of the coronary artery leads to ischemia and pain while permanent occlusion of the artery leads to transmural myocardial infarction (MI).

Myocardial revascularization has been an established mainstay in the treatment of CAD for almost half a century. Coronary artery bypass grafting (CABG), used in clinical practice since the 1960s, is arguably the most intensively studied surgical procedure ever undertaken. However, since coronary interventions have become much more in practice and multiple randomized controlled trials (RCTs) were conducted, these RCTs reported that CABG resulted in up to a five-fold reduction in the need for re-intervention, modest survival benefit or a survival benefit in patients above 65 years old and those with diabetes as compared to percutaneous treatment in patients with multi-vessel disease [4].

Postoperative atrial fibrillation (AF) is the commonest of all the known cardiac arrhythmias after cardiac surgery. It has both short- and long-term adverse impacts on patients, as it is known to be associated with prolonged intensive care unit (ICU) and total hospital stay [5]. It is also associated with increased frequency of reoperations, myocardial infarction, increased use of inotropes and intra-aortic balloon pump (IABP). Other adverse impacts of AF include postoperative acute respiratory distress syndrome (ARDS), cerebrovascular accidents (CVA), altered cognitive functions, surgical site infection, renal failure and gastrointestinal complications [6-8]. These all complications collectively lead to additional use of resources and hence increasing the cost of care [9]. Both short- and long-term survival rate is also reduced in patients who had AF after cardiac surgery as compared to those who did not have it [10,11].

Different studies identified various risk factors associated with atrial fibrillation after cardiac surgery. Common among them are patient’s age, preoperative cardiac arrhythmias, congestive heart failure (CHF), ischemic heart disease, use of preoperative IABP, and obesity [12,13]. Intraoperative risk factors include the number of distal anastomoses and the use of cardiopulmonary bypass [12,14]. Postoperatively prolonged ventilation is also known to be an independent risk factor for postoperative atrial fibrillation [4].

Impact of ethnicity upon the frequency of postoperative new-onset atrial fibrillation (NOAF) after cardiac surgery is an established factor [5,15] and most of these studies showed risks of postoperative new-onset AF after all cardiac surgeries. There is a paucity of data regarding the frequency of AF after isolated CABG and its risk factors in our geographic location. As compared to the western population, the South Asian population is demographically different, especially in terms of age and gender, which may lead to variation in CABG outcomes including atrial fibrillation. Since the exact burden is still unknown, in our geographical location, therefore, we aimed at determining the frequency of postoperative atrial fibrillation among patients undergoing isolated coronary artery bypass grafting. So that appropriate strategies could be made to combat the situation.

## Materials and methods

This prospective observational study was conducted on 163 consecutively selected patients undergoing first time isolated CABG at the Department of Cardiothoracic Surgery, Aga Khan University Hospital, Karachi, from October 2012 to March 2013. This study was approved by the ethical review board of Aga Khan University Hospital, Karachi (2557-Sur-ERC-13), and written informed consent was taken from all the enrolled patients. Patients with redo-sternotomy, preoperative atrial fibrillation, and with other cardiac pathology were excluded from the study. Diagnosis of CAD was made based on coronary angiography. As defined by the Society of Thoracic Surgeons (STS), postoperative atrial fibrillation/flutter (AF) is one which requires treatment. It does not include recurrence of AF which had been present preoperatively. Postoperative AF was defined in the patients with postoperative 12-lead electrocardiographic (ECG) finding of absence of P waves, replaced by unorganized electrical activity and irregular R-R intervals. The sample size for the study of 163 patients was calculated using World Health Organization (WHO) sample size calculator version 2.0, assuming 29% as the expected frequency (p) of postoperative atrial fibrillation among patients undergoing CABG [5], 7% margin of error, and 95% confidence level. Data were collected using a pre-defined structural proforma which included socio-demographic variables (age, gender, weight, and height), risk profile (hypertension, diabetes mellitus, smoking, ejection fraction, use of statins, and preoperative IABP requirement), perioperative variables (type of surgery, number of distal anastomosis, use of internal mammary artery, aortic cross-clamp time, cardiopulmonary bypass time), and postoperative variables (postoperative AF, timing of AF, use of inotropes, ventilator requirement, length of ICU stay). All the procedures have been done by the consultant cardiac surgeon with a minimum of five years of experience. Postoperative atrial fibrillation was assessed during the postoperative hospital stay. Data analysis was carried out using IBM SPSS Statistics for Windows, Version 21.0 (IBM Corp., Armonk, NY, USA). The continuous variables were expressed as mean ± standard deviation (SD) and the categorical variables were reported as frequency (percentage). Effect modifiers and confounding factors associated with atrial fibrillation were explored by stratified analysis and Chi-square test was applied to see the effect of these on postoperative AF. Statistical significance criteria were taken as p-value ≤ 0.05.

## Results

A total of 163 patients were enrolled as per inclusion and exclusion criteria. The demographic characteristics of the patients are presented in Table 1. Mean age was 58.66 ± 9.77 years ranging between 40 and 85 years with male predominance of 81% (132). Most common comorbidity was hypertension in about 68.1% (111) of the population, followed by diabetes mellitus. Majority of patients were overweight with the mean body mass index (BMI) of 26.88 ± 5.16 kg/m^2^, history of smoking was present in about half of patients. Most of the patients were admitted with normal ejection fraction and emergent CABG was performed in only 4.3% (seven) of patients. All patients had undergone coronary angiography prior to surgery, and the most common indication for CABG was three-vessel CAD in 83.4% (136) followed by two-vessel coronary artery disease (2VCAD). Baseline demographic characteristics, cardiac risk profile, and severity of CAD are presented in Table 1.

**Table 1:** Baseline demographic characteristics, cardiac risk profile, and severity of coronary artery disease (CAD).

Characteristics	Total [N = 163]
Age (years)	58.66 ± 9.77
Male gender	132 (81%)
Weight (kg)	72.117 ± 13.56
Height (cm)	172.46 ± 16.76
Body mass index (kg/m^2^)	26.88 ± 5.16
Diabetes	89 (54.6%)
Hypertension	111 (68.1%)
Smoking	70 (42.9%)
Ejection fraction (%)	51.38 ± 12.5
Number of coronary artery disease (CAD)	
Single vessel (SV) CAD	3 (1.8%)
Two vessels (2V) CAD	24 (14.7%)
Three vessels (3V) CAD	136 (83.4%)

All of the patients were operated for isolated CABG on cardiopulmonary bypass (CPB) by establishing an extracorporeal circulation. Mean CPB time was 94.15 ± 58.21 minutes and mean aortic cross-clamp time was 58.59 ± 22.56 minutes.

**Figure 1:**
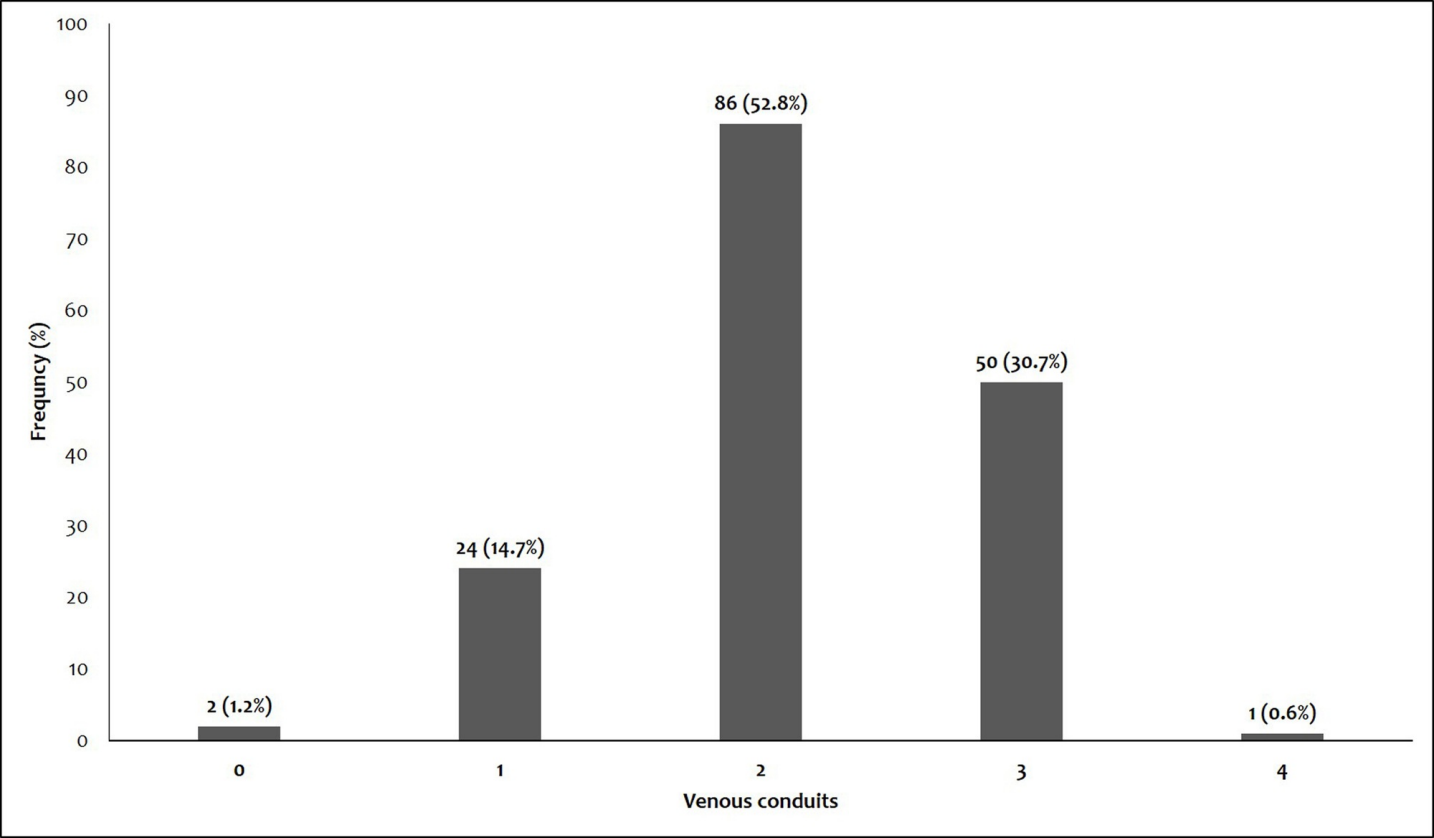
Distribution of venous conduits for distal anastomoses.

Seven patients required IABP which was inserted preoperatively because of the hemodynamic instability and low ejection fraction. Left internal mammary artery (LIMA) was the only arterial conduit which was used in 100% of patients for distal anastomosis between LIMA and left anterior descending artery (LAD). Great saphenous vein was harvested from leg to be used as a venous conduit for remaining distal anastomoses. 52.8% (86) of patients had two distal coronary anastomoses and 30.7% (50) of patients required four three anastomoses, as presented in Figure 1.

Postoperative AF was observed in 42 (25.8%) patients with the mean age of 61.29 ± 10.22 years. Most of them were overweight with a mean BMI of 27.04 ± 4.85 kg/m^2^, 76.2% (32) had a history of hypertension, diabetes mellitus was associated with 33.3% (14) patients with postoperative AF, mean ejection fraction was 50.6 ± 13.08% and 50.0% (21) of them were smokers. Distribution of coronary artery disease in patients with postoperative AF was observed as three-vessel coronary artery disease (3VCAD) in 83.3% (35), 2VCAD was present in 7.1% (three) and rest of 9.5% (four) patients had single vessel disease. IABP was required preoperatively in 7.1% (three) of patients with postoperative AF. Demographic and laboratory characteristics and surgical data by postoperative atrial fibrillation are presented in Table 2.

**Table 2:** Demographic and laboratory characteristics and surgical data by postoperative atrial fibrillation. P-values are based on t-test or Mann–Whitney U test for continuous variables and chi-square test for categorical variables. *Significant at 5% level of significance

Characteristics	Postoperative atrial fibrillation (AF)		P-value
	Yes	No	
	n = 42	n = 121	
Age (years)	61.29 ± 10.22	57.87 ± 9.46	0.049*
Body mass index (kg/m^2^)	27.05 ± 4.86	26.76 ± 5.25	0.753
Weight (kg)	70.83 ± 15.78	72.36 ± 12.6	0.528
Height (cm)	161.52 ± 9.78	176.34 ± 135.9	0.482
Postop ventilation time (hours)	22.38 ± 60.99	11.63 ± 11.81	0.065
Cross Clamp Time (minutes)	58.48 ± 24.39	58.7 ± 22.08	0.956
Ejection fraction (%)	50.6 ± 13.08	51.5 ± 12.28	0.687
Initial intensive care unit stay (hours)	42.75 ± 22.09	43.3 ± 21.25	0.886
Diabetes	33.3% (14)	62% (75)	0.001*
Hypertension	76.2% (32)	65.3% (79)	0.191
Smoking	50% (21)	40.5% (49)	0.283

## Discussion

Postoperative AF is the commonest complication after cardiac operations which has adverse impacts on patients in terms of worsening of postoperative state, prolonged hospitalization, and prolonged ICU stay and it is a predictor of advanced heart disease with worse prognoses [9,16,17]. The incidence of new-onset postoperative AF after cardiac surgery is rising and literature have reported the incidence rate of up to 40% in different studies and it has remained unchanged despite advancement in surgical strategy, perfusion techniques on cardiopulmonary bypass and postoperative care in presence of new anti-arrhythmic drugs.

The incidence of postoperative AF after isolated CABG is between 30 and 40%. This was much lower in our study population, i.e., 25.8%. One reason could be lower mean age of our operative population as compared to the data from the western population. This also shows that ischemic heart disease in population presents earlier however this will require further investigation. Patients with postoperative AF are at increased risk of developing CVA with an accumulative increased total cost of treatment [11]. We analyzed these predictors in our study but they did not achieve statistical significant value. Sezai et al. demonstrated some parameters related to postoperative AF which are the size of the left atrium, ejection fraction and advanced age [18]. Advanced age was an independent risk factor of postoperative new-onset AF [13,19,20]. These all factors were analyzed in this study and we did not find any significant relation with postoperative AF. Hwang et al. reported in their study that the majority of patients with postoperative AF were restored to sinus rhythm. It was also observed that restoration of atrial contractility was found after conversion to sinus rhythm in patients with postoperative AF [21]. Postoperative AF usually occurs within the first four postoperative days after cardiac surgery, most of them occur on the second postoperative day, and only six percent of patients experience AF after the sixth postoperative day [22]. This study has observed that in the majority of our patients, postoperative AF developed within the first 48 hours after isolated CABG. The mechanism of postoperative AF after cardiac surgery is different from those of nonsurgical patients, it is multifactorial in origin. Pericardial and systemic inflammation, excessive production of catecholamines, autonomic imbalance, and interstitial mobilization of fluid with resultant changes in volume, pressure, and neurohumoral environment during the postoperative period are some possible mechanisms of developing postoperative AF after cardiac surgery [20]. Majority of postoperative AF episodes end spontaneously even without taking any therapeutic measures. It has also been reported in the literature that the restoration rate to normal sinus rhythm was 93% to 98% in patients with postoperative AF [23]. The current study could not assess the restoration time after postoperative AF and treatment of postoperative AF as this study was designed to determine the frequency of postoperative AF after Isolated CABG. Current guidelines have recommended initiating anticoagulation therapy within 48 hours of the onset of AF because of a doubling of the risk of stroke [24]. Obesity is one of the predictors of postoperative AF, predominantly severe obesity which has an influence on cardiopulmonary physiology particularly left atrial enlargement is a common finding in obese patients [25]. This study population was overweight with BMI of 26.88 ± 5.16 kg/m^2^ but we could not find a statistically significant association between obesity and postoperative AF after isolated CABG.

Mariscalco et al. conducted a study of CABG at two Italian centers. They reported 31% of patients developed postoperative AF out of 1,832 patients who underwent isolated CABG. Postoperative AF was found in association with increased short- and long-term mortality. Their study also identified that embolic events were the main cause of death in patients with postoperative AF [26]. Villareal et al. reported the frequency of postoperative AF was 16% in patients who underwent first time isolated surgical revascularization while postoperative AF was associated with late mortality at Texas Heart Institute [11]. In the current study, the frequency of postoperative AF is comparable but there was single in-hospital mortality. Since the overall mortality in our study population was very low as described earlier, risk factors for mortality cannot be well studied. To study the role of postoperative AF as a predictor of early and late mortality, we need data of much larger size. As our database grows in the future, we will be able to analyze this relationship.

Mueller et al.’s study analyzed the risk factors of postoperative AF and identified that right coronary artery disease, age and diabetes are independent risk factors for postoperative AF [27]. We analyzed these variables as predictors of postoperative AF but the results did not achieve statistical significance although the number of patients with postoperative AF having diabetes mellitus was higher.

Postoperative AF can predispose to ventricular dilation and decrease cardiac output by impairing atrial systolic contraction [28]. Postoperative AF promotes decreased ventricular filling and circulatory stasis in the left atrium, making the patient susceptible to stroke and embolic events. Judicious use of thrombotic prophylaxis and correction electrolyte imbalances are recommended in the prophylaxis of postoperative AF [29].

This is the first prospective study which is looking for the frequency of postoperative AF after isolated CABG. No previous study has addressed this issue in this region before. However, the study does not adequately explore the predictors of postoperative AF as the study was designed only to measure the frequency of postoperative AF after isolated CABG. Since this point is of great clinical relevance we encourage studies designed to look for predictors.

## Conclusions

The frequency of postoperative atrial fibrillation in our study was found to be 25.9% which is comparable to world literature. An important finding that comes through this study is a younger population undergoing CABG which raises the possibility of early manifestation of ischemic heart disease in our region. This, however, needs further investigation. We were unable to point out the factors predictive of postoperative AF; studies with larger sample size would help in that regard.
